# Ultrasound in the Evaluation of Radial Neuropathies at the Elbow

**DOI:** 10.3389/fneur.2019.00216

**Published:** 2019-03-12

**Authors:** Ted G. Xiao, Michael S. Cartwright

**Affiliations:** Department of Neurology, Wake Forest School of Medicine, Winston-Salem, NC, United States

**Keywords:** ultrasound, radial nerve, entrapment, radial tunnel, clinical recommendation

## Abstract

There are five sites at which radial nerve entrapment at the elbow has been commonly reported. These include the level of the fibrous bands within the extensor carpi radialis brevis, the thickened fascial tissue at the radiocapitellar joint, the leash of Henry, the arcade of Frohse, and the distal border of the supinator muscle. This review describes the anatomy of the radial nerve at the elbow and the surrounding structures, and then provides an overview of the literature supporting the use of ultrasound to assist in the evaluation of suspected radial neuropathy at the elbow. This review concludes with a suggested ultrasonographic approach for the systematic evaluation of suspected radial neuropathy at the elbow.

## Introduction

The radial nerve is one of the major nerves of the upper extremity and one of the main branches of the brachial plexus. It innervates a number of muscles responsible for extending the elbow, wrist, and fingers and transmits sensation from the posterior surface of the arm, forearm, and hand. There are several sites around the elbow at which the radial nerve can be compressed, and the site of compression determines the clinical syndrome. Some of these named syndromes are controversial and overlap with each other. We prefer the broader and encompassing term, “radial neuropathy at the elbow,” and will use this term throughout this article unless it is clear that compression is at a discrete site in a specific case (such as the arcade of Frohse).

The overall goal of this article is to define the anatomy of the radial nerve at the elbow, clarify the potential sites of radial nerve compression at the elbow, describe the studies involving ultrasound of the radial nerve at this site, and outline a clinical neuromuscular ultrasound approach for evaluation of radial neuropathy at the elbow.

## Radial Nerve Anatomy

The radial nerve (which includes C5 to T1 nerve roots) is a terminal branch of the posterior cord of the brachial plexus, and it carries motor and sensory nerve fibers. The radial nerve arises in the axilla, posterior to the axillary artery. It enters the posterior compartment of the arm under the teres major muscle. Branches of the radial nerve innervate the long and lateral heads of the triceps brachii. The radial nerve travels over the shallow groove of the humerus (spiral groove) with the deep brachial artery. It wraps around the humerus laterally and branches off to supply the medial head of triceps brachii. The radial nerve travels between the brachialis and brachioradialis muscles anterior to the lateral epicondyle to innervate the brachioradialis and extensor carpi radialis longus muscles.

The radial nerve then travels distally into the forearm through the cubital fossa, a triangular region defined by the epicondyles superiorly, the pronator teres medially, and the brachioradialis laterally. It divides into two terminal branches, the superficial sensory branch and the deep motor branch.

The deep motor branch travels into the radial tunnel, a space spanning from the radiocapitellar joint to the distal edge of the superficial supinator muscle (1).

The tunnel is defined laterally by the mobile wad of Henry [brachioradialis, extensor carpi radialis longus, and extensor carpi radialis brevis (ECRB) muscles] and medially by the biceps tendons and brachialis. The tunnel is superficially bound by the radial recurrent vessels (leash of Henry), the superficial head of the supinator muscle, and the brachioradialis muscle. The capsule of the radiocapitellar joint and the deep head of the supinator muscle form the floor of the radial tunnel. The deep motor branch enters the radial tunnel underneath the fibrous bands of the extensor muscles in the forearm at the level of the radial head. It crosses the leash of Henry at the radial neck and beneath the tendinous margin of the ECRB. The deep motor branch innervates the ECRB and supinator. Lastly, the nerve exits the radial tunnel beneath the aponeurotic margin between the superficial and deep layers of the supinator muscle as the posterior interosseous nerve (PIN). The PIN travels distally along the interosseous membrane between the radius and ulna, supplying all the extensor muscles on the dorsal surface of the forearm, except the anconeus, brachioradialis, and extensor carpi radialis longus (which are innervated by the radial nerve proper).

The superficial sensory branch of the radial nerve travels laterally with the radial artery and beneath the brachioradialis. Once it passes between the tendons of the brachioradialis and extensor carpi radialis longus, the superficial branch pierces the fascia and divides into two branches to supply sensory innervation to the posterior skin of the lateral three and a half digits.

## Radial Neuropathies at the Elbow

There are two ways in which radial neuropathy at the elbow can be conceptualized. The first is based on the anatomic location of the lesion within the radial nerve, which is discussed below. The second is based on the clinical syndrome, which are presented in Table 1.

**Table 1:** This table includes the named clinical syndromes that arise from radial mononeuropathies at the elbow.

**Clinical syndrome**	**Site of pathology**	**Symptoms**	**Signs**
Radial tunnel syndrome	The motor branch of the radial nerve within the radial tunnel	Lateral proximal forearm pain 3 to 4 cm distal to lateral epicondyle in the area of the mobile wad and radial tunnel	Pain with resisted active supination or wrist extension; pain with resisted middle finger extension; localized tenderness along the path of the motor branch of the radial nerve
PIN or supinator syndrome	The PIN at the arcade of Frohse	Pure motor symptoms, weakness in forearm and hand	Weakness in wrist and finger extension, radial deviation with attempted wrist extension
Lateral epicondyle syndrome	Lateral epicondyle at insertion of the ECRB	Pain with resisted wrist extension; pain with gripping motion	Focal point tenderness on the lateral epicondyle at insertion of the ECRB; exacerbated pain with resisted wrist extension with fully extended elbow, resisted extension of long fingers, and passive wrist flexion in protonation

*In addition, lateral epicondyle syndrome is included, as it may present with similar symptoms to radial mononeuropathies. ECRB, extensor carpi radialis brevis; PIN, posterior interosseous nerve*.

The first location of potential radial mononeuropathy at the elbow is the floor of the radial tunnel, which is the fascial tissue of the radiocapitellar joint. Thickening of this tissue can induce entrapment of the radial nerve (2). Compression can also occur due to osteoarthritis or synovitis of the radiocapitellar joint (1). The second location is within the radial tunnel caused by hypertrophic crossing branches of the leash of Henry (3). The third location is the proximal and medial edge of the ECRB. The proximal tendinous arch of the ECRB and the aponeurosis of the undersurface of the ECRB are both suspected to contribute to possible radial nerve compression at this site (4). The fourth location (and likely the most common entrapment location) is the aponeurotic margin between the superficial and deep layers of the supinator muscle, often referred to as the arcade of Frohse (5). The fifth described location of radial entrapment neuropathy at the elbow is at the distal border of the supinator muscles. Compression at this site is intermittent due to pressure from the distal edge of the radial head (1).

## Ultrasound for Radial Neuropathies at the Elbow

Neuromuscular ultrasound has been used to localize and visualize lesion sites, classify severity, and detect potential causes of mononeuropathies (6, 7). Measurement of nerve cross-sectional area with ultrasound is an accurate parameter for the diagnosis of carpal tunnel syndrome (8) and has been shown to significantly increase diagnostic sensitivity in ulnar neuropathy at the elbow when combined with electrodiagnosis (9). For the less frequently studied radial neuropathies at the elbow, neuromuscular ultrasound has shown benefit in case reports and series.

Neuromuscular ultrasound was used to establish reference ranges of cross-sectional area of the radial nerve at the antecubital fossa, which range between 4.5 mm^2^ and 14.3 mm^2^ (10). While the cross-sectional area is the preferred method for measuring nerve size, early studies reported results in nerve diameter, with the deep branch of the radial nerve ranging between 1.30 mm and 2.13 mm (11). Based on reference ranges of the radial nerve, neuromuscular ultrasound case reports and series have consistently identified nerve enlargement in radial neuropathies at the elbow in comparison to the contralateral arm and healthy controls (11–17). Additional characteristic ultrasound findings of an injured nerve that may assist in localization include decreased echogenicity, enlarged nerve fascicles, and increased nerve vascularity (13, 18–20). Given the variability in reported reference values, side-to-side ultrasonographic comparison of nerve cross-sectional area is often helpful in suspected unilateral cases of radial neuropathy at the elbow.

The ability to distinguish nerve from surrounding tissues (21) and demonstrate nerve morphology and dimensions (22) allow ultrasound to provide insight when electrodiagnostic studies are not conclusive, and ultrasound can also assist in identifying the underlying etiology (19, 23, 24). Electrophysiologic studies can define the severity of an entrapment injury, but often cannot identify the underlying cause of the mononeuropathy because they do not typically assess the surrounding structures. Numerous cases have utilized ultrasonography to identify various radial neuropathies at the elbow. Further, visualization of the surrounding anatomy at the site of lesion allows for the identification of the underlying causes of radial neuropathies. These reported etiologies include ganglion cysts (25–27), masses/tumors (28–30), entrapment from surrounding tissues (31), vascular structures (3), and iatrogenic causes from interventions (32, 33).

Interestingly, neuralgic amyotrophy (also known as Parsonage-Turner syndrome), has been redefined over the past several years, with new knowledge coming from ultrasonographic studies of the radial nerve (34–36). Neuralgic amyotrophy used to be described as a brachial plexopathy, but ultrasound and other imaging studies have shown that the majority of pathologic changes actually occur outside the brachial plexus, with several studies showing radial nerve abnormalities. The most commonly described nerve imaging abnormalities in this condition include hourglass deformities of the nerve. Detailed imaging has shown fascicles rotating around each other, which suggests nerve torsion, and, surgical exploration has confirmed nerve torsion in those cases (34). Further investigation is needed, but imaging findings now suggest that neuralgic amyotrophy is not a brachial plexopathy, but rather a process that involves motor-predominant peripheral nerves, and sometimes results in torsion of these nerves.

## Clinical Recommendations

Given the challenges with electrodiagnostic studies in suspected radial mononeuropathies (37), neuromuscular ultrasound provides a convenient and complementary tool to potentially diagnose the specific etiology causing the radial neuropathy at the elbow. In addition, numerous studies and case reports already mentioned have integrated ultrasound findings with the clinical and electrical diagnosis of radial neuropathies at the elbow. Based on the literature review and clinical experiences, ultrasound findings detected in radial mononeuropathies include focal nerve enlargement, nerve hypoechogenicity, enlarged nerve fascicles, and increased nerve vascularity (20). Using these parameters, we outline a systematic approach to identify the site and potentially the etiology of radial neuropathies at the elbow with ultrasound. Other detailed resources are available to assist readers in further understanding approaches to imaging the radial nerve at the elbow and identifying key surrounding structures (38, 39).

**Figure 1:**
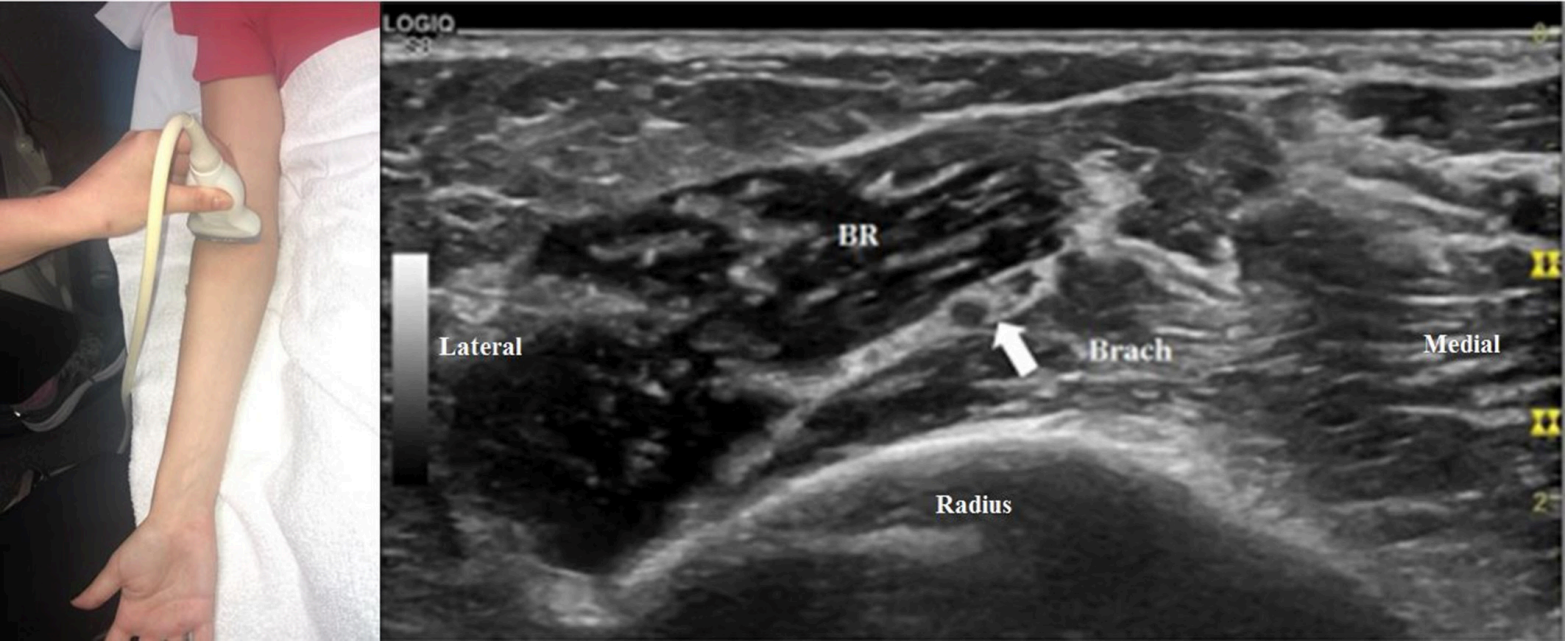
The transducer is placed just proximal to the right antecubital fossa to obtain a cross-sectional view. The radial nerve can be seen (arrow), with two large fascicles that will become the motor and sensory branches. In this image, the lateral fascicle will become the deep branch of the radial nerve and the medial fascicle will become the superficial branch. All images in this article were obtained from a volunteer specifically for illustrative purposes related to this article. Consent was provided. Brach, brachialis, BR, brachioradialis.

**Figure 2:**
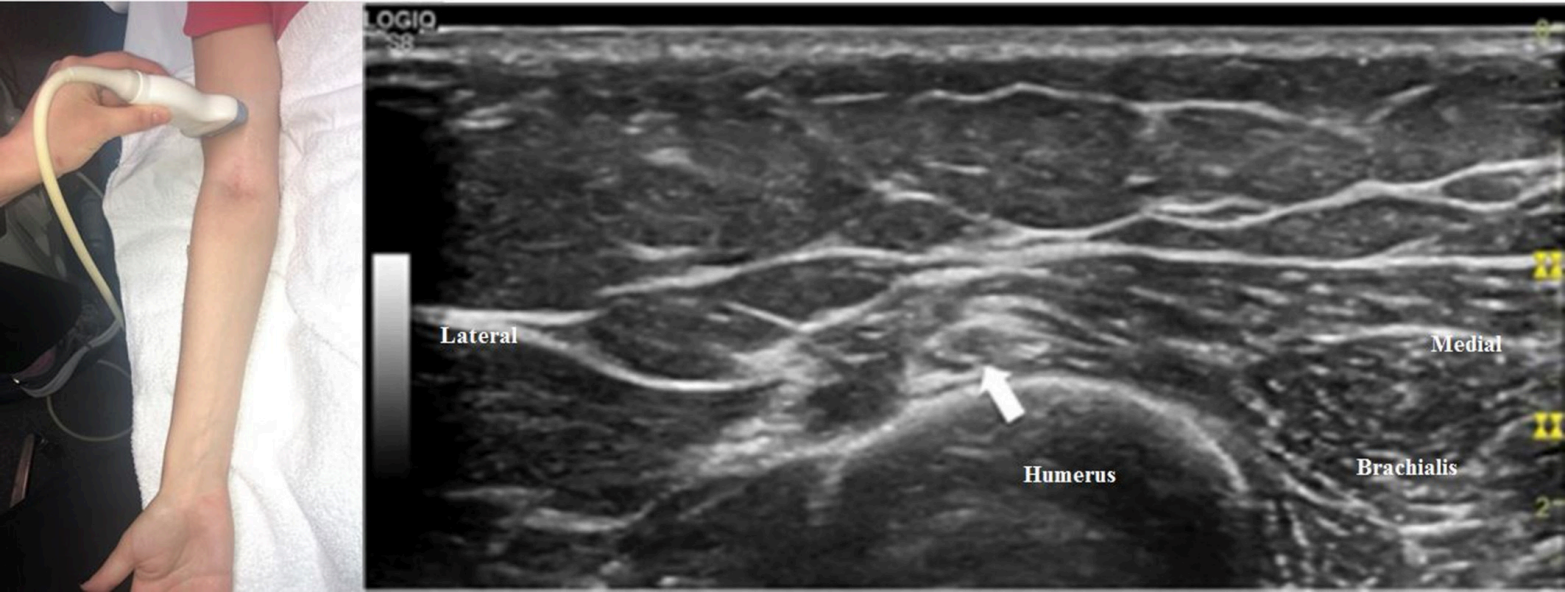
The transducer has been moved proximally compared to Figure 1, and the radial nerve (arrow) can now be seen adjacent to the humerus (the convex, hyperechoic structure just deep to the nerve) in the spiral groove.

The radial nerve bifurcates into the superficial radial sensory nerve and the deep motor nerve at the antecubital fossa. When the transducer is placed at the antecubital fossa to obtain a cross-sectional view (Figure 1), both the sensory and motor branches of the radial nerve are visualized between the brachialis and brachioradialis muscles. At this site (or within 1 cm proximally and distally), the ultrasonographer can assess for fibrous bands anterior to the radiocapitellar joint, the recurrent radial vessels of the leash of Henry, and the proximal edge of the ECRB. Moving the transducer proximally (Figure 2), the radial nerve can be identified adjacent to the humerus, coursing through the spiral groove between the long head of the triceps and the humerus. Near the spiral groove, the deep brachial artery can be identified adjacent to the radial nerve. If necessary, the radial nerve can be traced all the way proximally to the terminal branch of brachial plexus.

**Figure 3:**
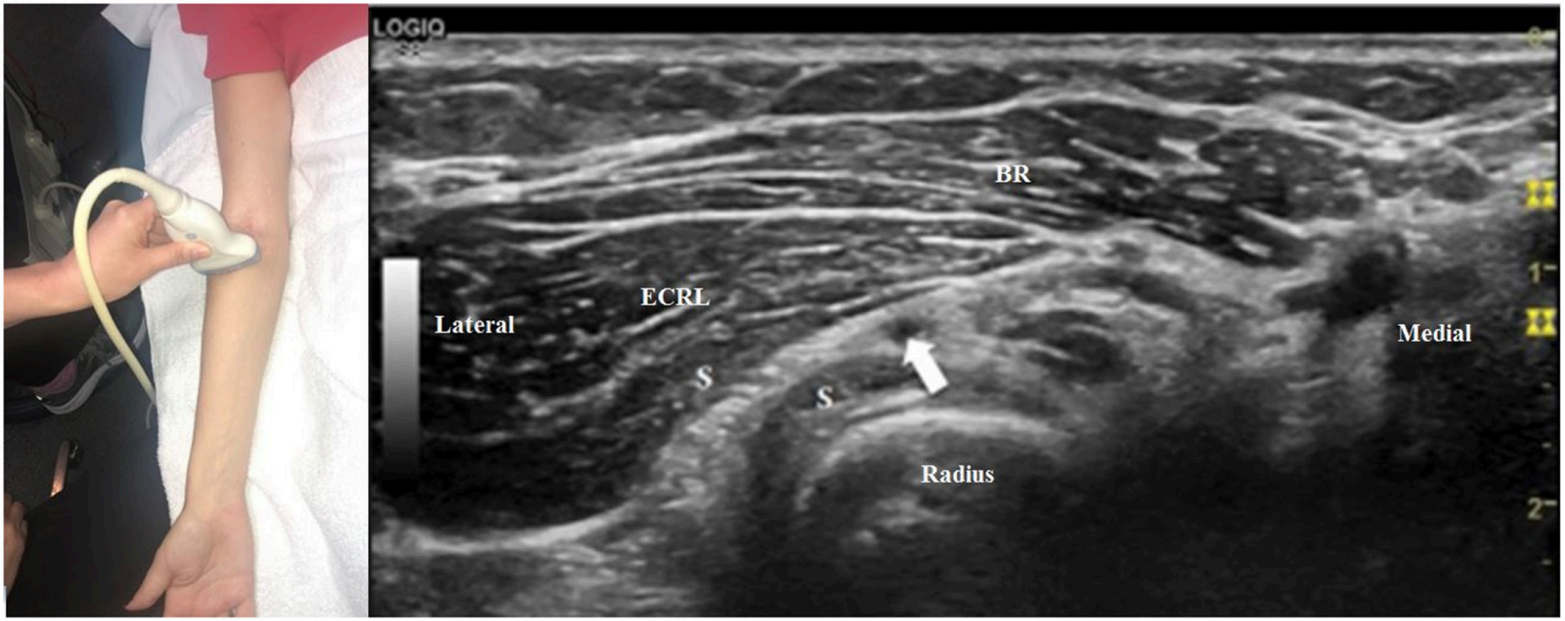
The transducer is now just distal to its position in Figure 1. The motor branch of the radial nerve (arrow) is just beginning to enter the arcade of Frohse, between the two layers of the supinator muscle (S). BR, brachioradialis; ECRL, extensor carpi radialis longus.

**Figure 4:**
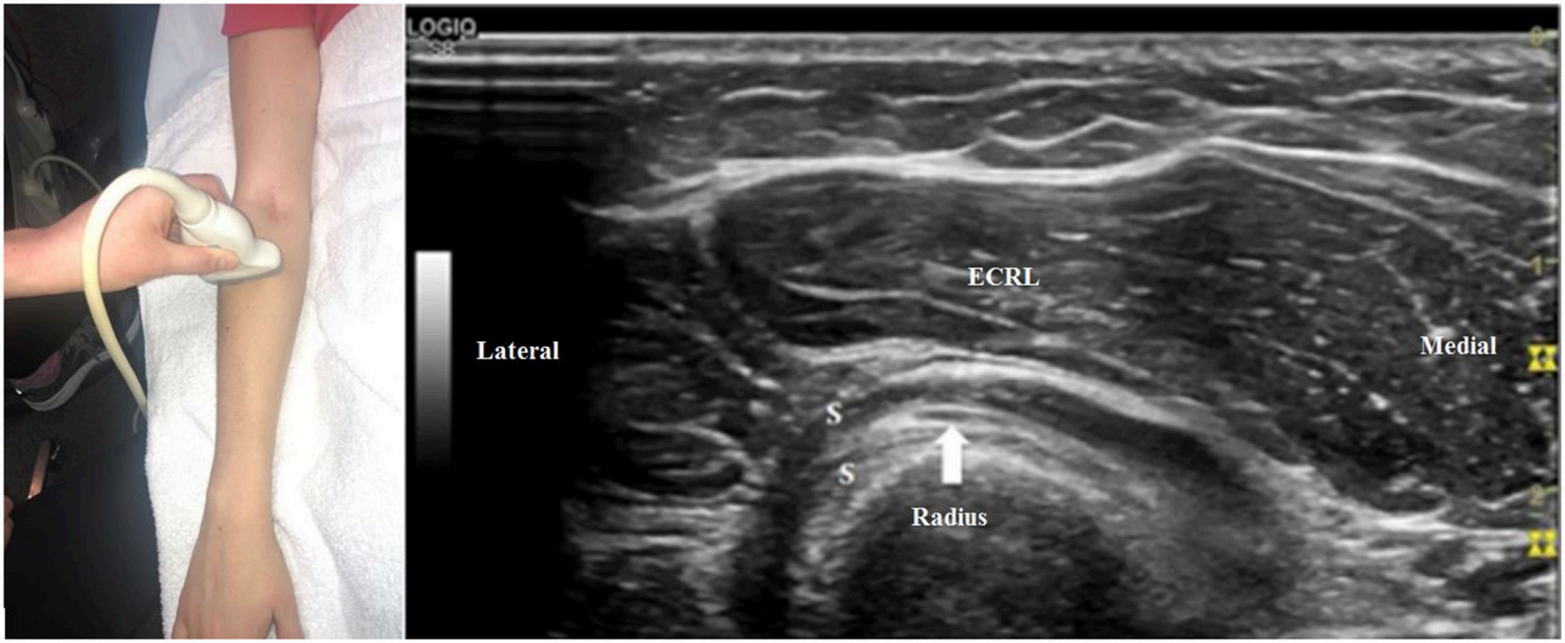
The transducer is just distal to its position in Figure 3. The motor branch of the radial nerve has now become the posterior interosseous nerve (arrow) and is imaged in a slightly oblique view between the two layers of the supinator (S). ECRL, extensor carpi radialis longus.

To search for a more distal nerve injury, start at the initial antecubital location from Figure 1. As one moves distally, the deep motor branch dives into the arcade of Frohse between the two layers of the supinator muscle (Figure 3). Moving the transducer further distally, the deep motor branch of the radial nerve exits the tunnel as the PIN. Figure 4 shows the PIN coursing obliquely to the posterior forearm around the lateral aspect of the radius between the layers of the supinator muscle. It courses on the interosseous membrane and in front of the extensor pollicis longus until it divides to innervate the individual muscles of the radial and dorsal surface of the forearm.

**Figure 5:**
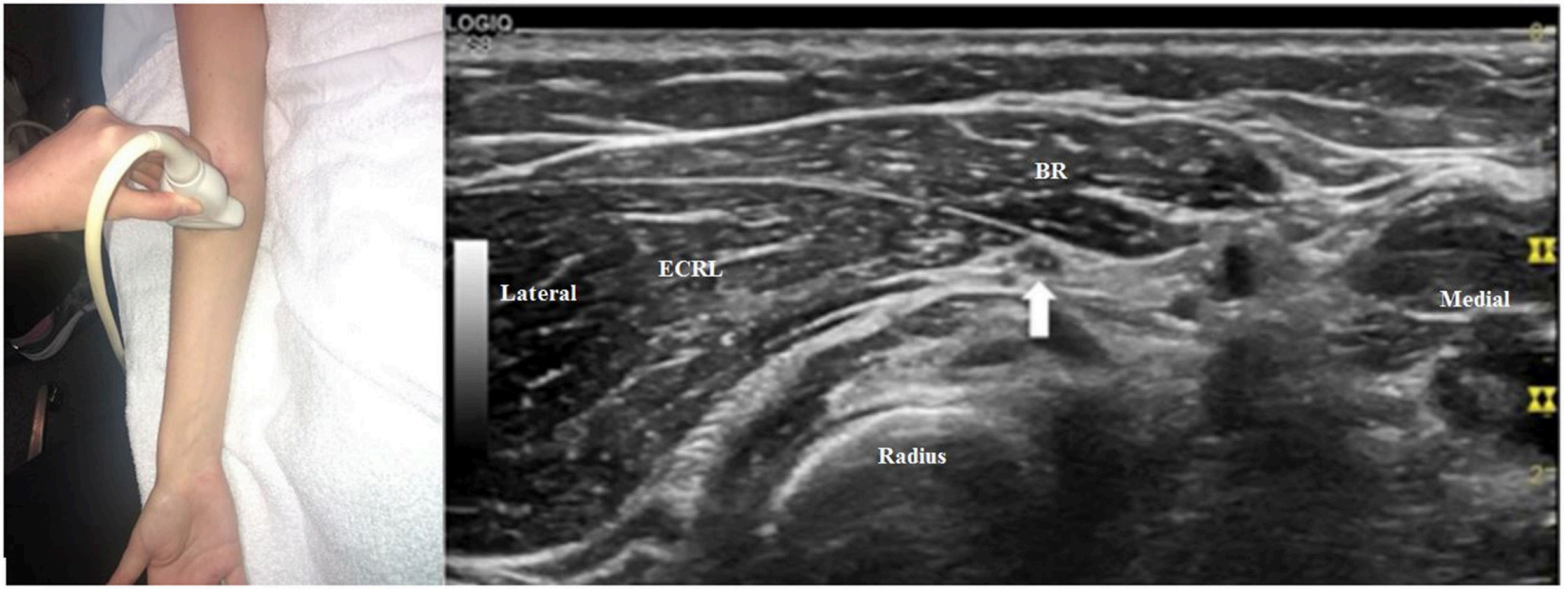
The transducer is just distal to the antecubital fossa and the superficial radial sensory branch of the radial nerve can be seen (arrow). BR, brachioradialis; ECRL, extensor carpi radialis longus.

**Figure 6:**
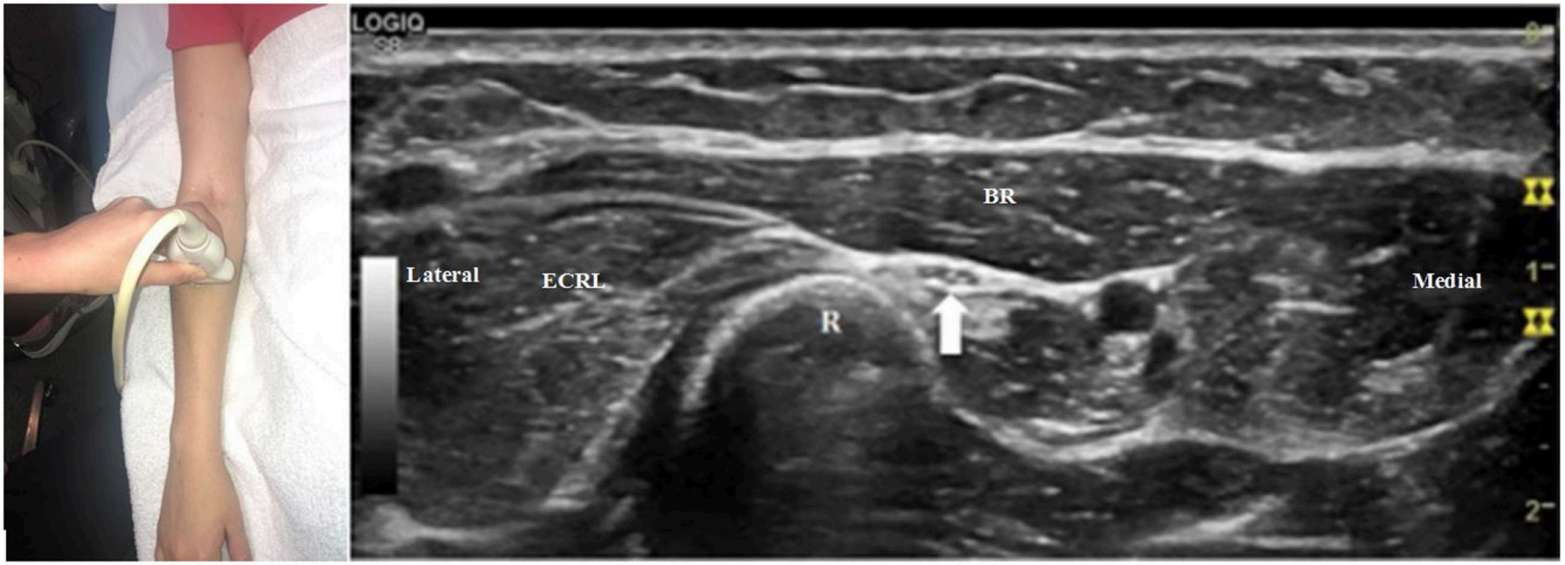
The transducer is now more distal than in Figure 5, and the superficial radial sensory nerve (arrow) is seen next to the convex, hyperechoic radius (R). BR, brachioradialis, ECRL, extensor carpi radialis longus.

To track the superficial sensory branch of the radial nerve, start again in the antecubital fossa and move the transducer distally (Figure 5). The superficial radial sensory nerve travels lateral to the radial artery beneath the brachioradialis muscle and tendon (Figure 6). The nerve travels between the brachioradialis and extensor carpi radialis longus tendons. Eventually, the superficial radial nerve pierces the overlying forearm fascia and divides into lateral and medial divisions and further divides into dorsal digital nerves (40).

As the radial nerve, deep motor branch, PIN, and/or superficial radial branch are scanned, it is important to note the cross-sectional area, overall echogenicity, echotexture, and fascicle size and distribution. At areas of focal nerve enlargement, the cross-sectional area of the nerve should be measured using the trace function of the ultrasound device and tracing along the inner border of the hyperechoic epineurium. This can then be compared to the contralateral side in individuals with unilateral symptoms. In addition, the nerve at the site of interest should be examined using Doppler to determine if there is increased blood flow within the nerve (41). Finally, it is important to view the site of interest in at least two orthogonal planes, and sometimes even more imaging planes are needed. Images from the site of interest should be saved for clinical care and documentation purposes.

**Figure 7:**
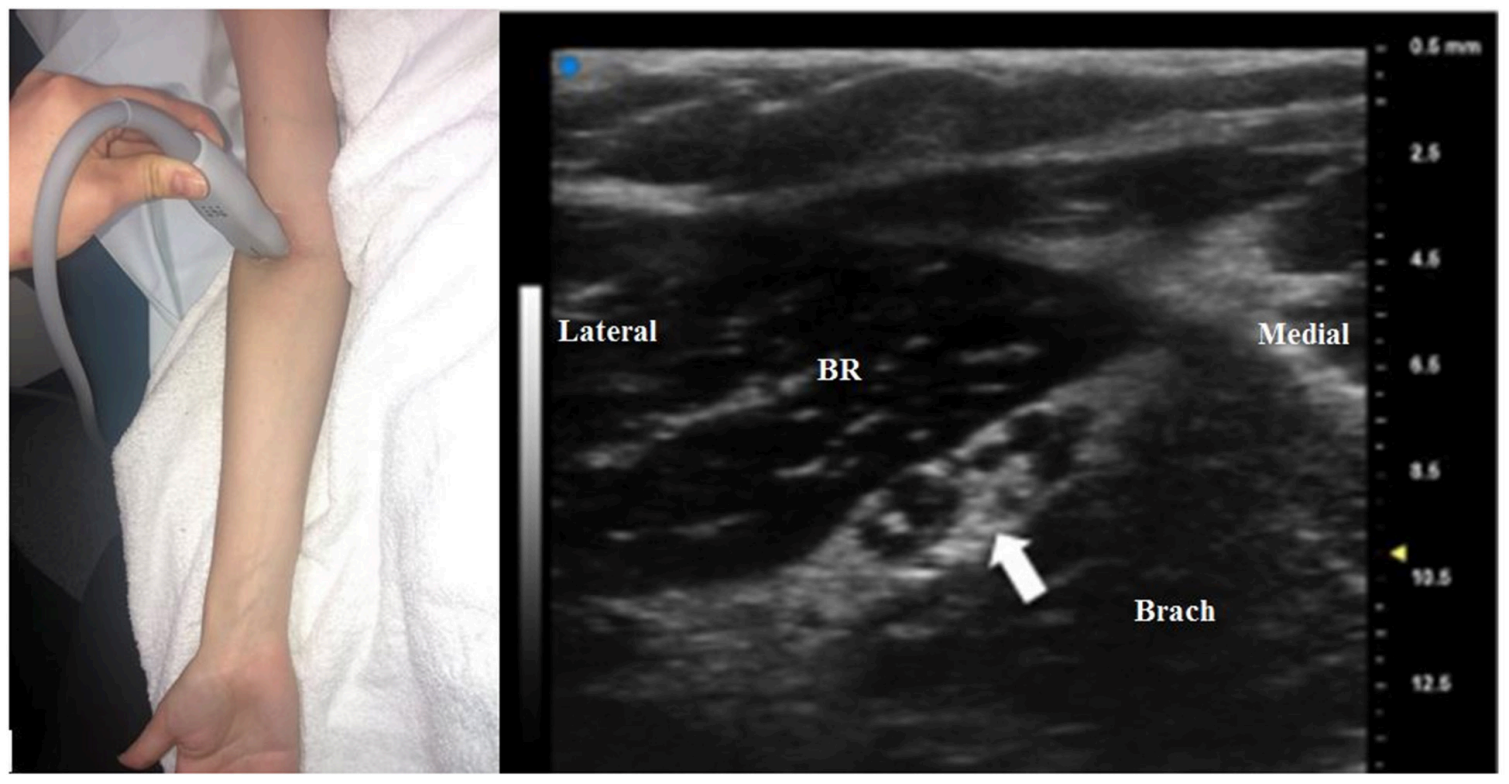
An ultra-high frequency 48 MHz transducer is used to image the radial nerve (arrow) just proximal to the antecubital fossa. Two prominent groups fascicles can be seen within the radial nerve, and the lateral group of fascicles will become the deep branch of the radial nerve and the medial fascicles will be become the superficial branch. This matches with the site of Figure 1, but the fascicular structure of the nerve can be seen in more detail. BR, brachioradialis; Brach, brachialis.

Very high-frequency ultrasound (VevoMD with a 48 MHz transducer, FujiFilm, Bothell, WA, USA), if available, can demonstrate the highly detailed anatomy of the radial nerve (Figure 7), which may assist in diagnosis. While this technology is not currently universally available, it is becoming more commonplace and in our opinion may be part of the imaging standard in the near future.

## Conclusions

Radial neuropathy at the elbow involves a heterogeneous group of conditions in which the radial nerve proper or branches of the radial nerve (including the superficial sensory, deep motor, and PIN) may be involved. There are 5 sites at which entrapment can occur, and the underlying causes of radial mononeuropathies include cysts, vessels, trauma, or iatrogenic injuries that can be identified with ultrasound. A systematic approach to scanning the radial nerve with ultrasound can assist in the diagnosis and identification of these conditions. Future studies may involve evaluation of poorly understood conditions such as neuralgic amyotrophy and the use of ultra-high frequency ultrasound.

## Ethics Statement

We confirm that we have read the Journal's position on issues involved in ethical publication and affirm that this report is consistent with those guidelines.

## Author Contributions

MC contributed to the original idea, manuscript writing, editing, and proofreading. TX contributed to the literature search, manuscript writing, editing, and proofreading.

### Conflict of Interest Statement

The authors declare that the research was conducted in the absence of any commercial or financial relationships that could be construed as a potential conflict of interest.
